# Spleen Toxicity of Organophosphorus Flame Retardant TDCPP in Mice and the Related Mechanisms

**DOI:** 10.3390/toxics11030231

**Published:** 2023-02-27

**Authors:** Lanqin Cao, Lai Wei, Qiaoyun Du, Ying Su, Shuzi Ye, Kaihua Liu

**Affiliations:** 1 Xiangya Hospital, Central South University, Changsha 410078, China; 2 Xiangya School of Public Health, Central South University, Changsha 410078, China; 3 Department of Anatomy and Cell Biology, Carver College of Medicine, University of Iowa, Iowa City, IA 52242, USA

**Keywords:** TDCPP, splenic injury, NF-κB pathway, mitochondrial apoptotic pathway, chemokines and receptors, immunosuppression

## Abstract

Tris(1,3-dichloro-2-propyl) phosphate (TDCPP) is an organophosphorus flame retardant that has been utilized in recent years as a primary replacement for polybrominated diphenyl ethers (PBDEs) in a wide variety of fire-sensitive applications. However, the impact of TDCPP on the immune system has not been fully determined. As the largest secondary immune organ in the body, the spleen is considered to be an important study endpoint for determining immune defects in the body. The aim of this study is to investigate the effect of TDCPP toxicity on the spleen and its possible molecular mechanisms. In this study, for 28 consecutive days, TDCPP was administered intragastrically (i.g), and we assessed the general condition of mice by evaluating their 24 h water and food intake. Pathological changes in spleen tissues were also evaluated at the end of the 28-day exposure. To measure the TDCPP-induced inflammatory response in the spleen and its consequences, the expression of the critical players in the NF-κB pathway and mitochondrial apoptosis were detected. Lastly, RNA-seq was performed to identify the crucial signaling pathways of TDCPP-induced splenic injury. The results showed that TDCPP intragastric exposure triggered an inflammatory response in the spleen, likely through activating the NF-κB/IFN-γ/TNF-α/IL-1β pathway. TDCPP also led to mitochondrial-related apoptosis in the spleen. Further RNA-seq analysis suggested that the TDCPP-mediated immunosuppressive effect is associated with the inhibition of chemokines and the expression of their receptor genes in the cytokine–cytokine receptor interaction pathway, including four genes of the CC subfamily, four genes of the CXC subfamily, and one gene of the C subfamily. Taken together, the present study identifies the sub-chronic splenic toxicity of TDCPP and provides insights on the potential mechanisms of TDCPP-induced splenic injury and immune suppression.

## 1. Introduction

Tris(1,3-dichloro-2-propyl) phosphate (TDCPP) is one of the commonly used organophosphorus flame retardants (OPFRs) in a broad range of products, including furniture, buildings, textiles, cars, clothing, and other items [1]. Some polybrominated diphenyl ethers (PBDEs), such as Penta- and Octa-BDE, have been restricted or banned in the EU due to overwhelming evidence that they are environmentally persistent organic pollutants (POPs) [2,3,4]. With its widespread use, TDCPP has been universally distributed to the environment—in the atmosphere, soil, surface water, groundwater and even glaciers that migrate long distances to the poles [1,5]. Besides its prevalence in the environment, TDCPP and its metabolites have also been found in human biological samples such as placenta, breast milk, plasma, and urine in recent years [6,7]. Furthermore, emerging evidence suggests that TDCPP exposure can cause neurological, reproductive, endocrine, hepatic and renal damage [1,8,9]. Notably, it was listed as a known carcinogen on the Proposition 65 list of substances [10,11].

A number of studies have shown that TDCPP induces cytotoxicity through the activation of the mitochondrial apoptosis pathway [12]. Meanwhile, there is also evidence suggesting that TDCPP can induce neuronal damage by triggering the microglia-mediated inflammatory response [13] and secretion of inflammation-like adipokines [14]. In addition, it was demonstrated that TDCPP can disrupt the phagocytosis of human THP-1-derived macrophages through immunosuppression and promote cytokine/chemokine secretion and inflammatory responses [15]. 

The spleen, a secondary lymphoid organ with hematological and immunological activities, is the body’s largest immune organ. It has the immunological function of identifying pathogens and aberrant cells and removing them; hence, it plays a significant role in the innate and adaptive immune response [16]. Patients treated clinically with splenectomy have a reduced clearance of malaria-hosted red blood cells and a higher risk of other infectious diseases [17]. Therefore, the spleen, as a key immune organ, stores many immune cells and is often regarded as an endpoint for assessing immunotoxin injury [18].

Despite the advances in TDCPP research over the past few years, there is still a lack of adequate understanding of the splenic immunotoxicological properties of this emerging contaminant. Transcriptome analysis techniques are currently an important research tool for conducting toxicity explorations of emerging contaminants. In this study, we identified differential genes based on a transcriptome sequencing platform to provide a basis for understanding the mechanisms of TDCPP-induced inflammatory responses and immune damage in the mice spleen.

## 2. Materials and Methods

### 2.1. Animals and Exposure

Male, 4-week-old C57BL/6J mice (initial weight: 18 ± 2 g) were purchased from the Hunan SJA Laboratory Animal Co., Ltd. (SCXK(XIANG)2019-0004). All animal experiments were approved by the Laboratory Animal Welfare Ethical Committee of Central South University (Ethics Approval Code: XYGW-2021-113). The mice were housed in a climate control and pathogen-free room (6 animals/cage, 12 h dark–light cycle, 24 ± 2 °C, 65 ± 25% humidity). 

After at least 1 week of acclimatation, animals were randomly allocated into two groups using random number tables: the control and the TDCPP groups. The TDCPP group was intragastric administrated (i.g) with TDCPP (purity: 95%; Bide Pharmaceutical Technology Co., Ltd., Shanghai, China) of 300 mg/kg a day for 28 consecutive days. Corn oil was used in this study as the TDCPP solvent. TDCPP had a reported oral LD50 of 2670 mg/kg in male mice [19]. A rather high dose of 300 mg/kg (approximately 1/10 LD50) was used as the oral dose in this investigation to examine the subacute toxic effects. The 24 h water and food intakes on the 7th, 14th, 21st and 28th days of exposure were measured. Every three days during the same period, the weight of the mice was measured.

### 2.2. Tissue Collection

Anesthesia was induced in mice using sodium pentobarbital (i.p. 40 mg/kg). Peripheral blood sampling was performed after the mice were anesthetized. Upon tissue harvest, the spleen was divided into two pieces. One piece was instilled with cold 0.9% saline, fixed in 4% paraformaldehyde for 4 h at 4 °C and stored for further processing. The other piece of spleen was flash frozen in liquid nitrogen and stored at −80 °C until further processing. 

### 2.3. Hematoxylin-Eosin (H&E) Staining

All specimens underwent 4% paraformaldehyde fixation at 4 °C for 4 h before being moved to an ethanol gradient for dehydration. Dehydrated samples were then placed in a mixed solution of xylene and paraffin for paraffin permeation, followed with dewaxing with xylene and ethanol gradients in turn. After soaking and staining with hematoxylin and eosin dye, respectively, ethanol, xylene and neutral resin were used to dehydrate and seal the sections and make them transparent. An OLYMPUS BX53 digital camera and the DP73 controller software were used to take all of the digital pictures (×200). According to the standard classification, normal (0), mild (1), moderate (2), severe (3), and very severe (4) categories were used to determine the degree of splenic tissue damage. Six visual fields/mouse pathology scores were used to compute the severity of the damage for each group of at least three mice. The specific operation is based on the methods used in a prior investigation [20].

### 2.4. Immunohistochemistry (IHC) Staining

During the IHC staining of spleen tissue, the sample was paraffin-embedded, sectioned, and dewaxed in the same manner as the previous H&E staining. Then, the sections were heated in a microwave oven for antigen retrieval. Prior to the incubation of the primary antibody, sections were immersed in 3% hydrogen peroxide solution for 15 min at room temperature and incubated in 1% BSA for 15 min at room temperature to block. Primary antibody solutions at different dilutions were prepared and incubated overnight at 4 °C in humid boxes; then, the appropriate secondary antibody was added, and the mixture was left to sit at room temperature for 60 min in the dark. This was followed by DAB coloring, counterstaining with hematoxylin, dehydration with ethanol, and making it transparent with xylene, and resin that is neutral was used for sealing. Finally, under a 400-fold microscope (OLYMPUS, Tokyo, Japan, BX53 & DP73), at least three regions of the spleens of each group of mice were observed and statistically analyzed. The intensity of the stained signals was measured and analyzed using Image-Pro Plus 6.0 image analysis software (Media Cybernetics, Inc. Silver Spring, MD, USA) according to previous studies and the average density of the digital images was calculated [21]. The mean density of the digital images (×400) was designated as representative NF-κB/IFN-γ/TNF-α/IL-1β staining intensity (indicative of relative NF-κB/IFN-γ/TNF-α/IL-1β expression levels). All picture capture sessions used the same microscope settings, and the experimenters were unaware of the treatment groups in order to perform image quantification.

Antibodies used in this study: anti-NF-κB p65 (Wanleibio, China, WL01980), anti-IFN-γ (Wanleibio, China, WL02440), anti-TNF-α (Wanleibio, China, WL01896), anti-IL-1β (Wanleibio, China, WLH3903), and HRP Goat Anti-Rabbit IgG (H + L) (thermoFisher, Waltham, MA, USA, #31460).

### 2.5. Terminal dUTP Nick-End Labeling (TUNEL) Assay

The principle of the TUNEL assay is that, when apoptosis occurs, some DNA endonucleases will be activated. These endonucleases will cut off genomic DNA between nucleosomes. When genomic DNA breaks, exposed 3′-OH can be added with fluorescein dUTP under the catalysis of terminal deoxynucleotidyl transferase (TdT), Td, which can be detected with a fluorescence microscope and through flow cytometry. The specific operation was carried out according to previous research methods [22]. To quantify the image (×400) of the final spleen tissue, unified microscope settings (OLYMPUS, Tokyo, Japan, BX53 & DP73) were maintained in all image acquisition processes, with an excitation wavelength of 450 nm and emission wavelength of 520 nm. The spleen apoptosis of each group of three animals was examined for quantitative analysis by TUNEL assay as follows: The number of apoptotic cells and the total number of cells in at least three fields of view were counted for each animal, and the apoptotic rate in the three fields of view was calculated and the mean value was taken. The apoptosis rate was calculated according to the following formula: Apoptosis rate = number of apoptotic cells/total number of cells.

### 2.6. Western Blot (WB) Analysis

The radio immunoprecipitation assay (RIPA) strong lysate (Beyotime Biotechnology, Shanghai, China, P0013B) was used to lyse the spleen tissues for 30 min, after which they were centrifuged at 12,000× *g* for 15 min at 4 °C. The quantification of protein concentrations was performed using a BCA protein concentration detection kit (Beyotime Biotechnology, Shanghai, China, P0010). The tissue proteins were first incubated to primary antibodies at 4 °C overnight, and then to matching secondary antibodies for 1 h, and finally they were incubated using the LumiBest ECL substrate solution kit for visualization (ShareBio, Shanghai, China, SB-WB011). The protein band intensity was measured using Image-J software.

Antibodies used in this study: anti-TNF-α (Immunoway, Suzhou, China, YT4689, 1:1000), anti-IL-1β (Beyotime Biotechnology, Shanghai, China, AF7209), anti-NF-κB p65 (ABclone, Wuhan, China, A2547, 1:1000), anti-IFN-γ (Immunoway, Suzhou, China, YT2279, 1:1000), anti-Caspase-3 (Proteintech, Wuhan, China, 19677-1-AP, 1:1000) anti-Bcl-2 (ABclone, China, A19693, 1:1000), anti-Bax (Proteintech, China, 50599-2-lg, 1:1000), anti-Cytochrome C (Proteintech, China, 10993-1-AP, 1:1000), anti-β-actin (MultiSciences, Hangzhou, China, #HA-R1207-1-200, 1:1000), HRP Goat Anti-Rabbit IgG (H + L) (Abclone, China, #AS014, 1:4000), and HRP Goat Anti-Mouse IgG (H + L) (Abclone, China, #AS003, 1:4000).

### 2.7. RNA Extraction and cDNA Library Generation

Total RNA was isolated from each group of two mice spleen tissues, 100 mg/spleen tissue, using the TRIzol^®^ Reagent (Magen) in accordance with the manufacturer’s instructions. The Nanodrop ND-2000 system from Thermo Scientific (Waltham, MA, USA) was used to measure RNA concentrations based on the A260/A280 absorbance ratio, and the Agilent Bioanalyzer 4150 system was used to determine the RNA integrity number (RIN) (Agilent Technologies Inc., Santa Clara, CA, USA). Only approved samples were utilized to build the library. Following the manufacturer’s instructions, paired-end libraries were created using an ABclonal mRNA-seq Lib Prep Kit (ABclonal, Wuhan, China). Using oligo (dT) magnetic beads and divalent cations at high temperatures in ABclonal First Strand Synthesis Reaction Buffer, the mRNA was isolated from 1 μg of total RNA. Then, using mRNA fragments as templates, first-strand cDNAs were created using random hexamer primers and Reverse Transcriptase (RNase H), and second-strand cDNAs were created using DNA polymerase I, RNAseH, buffer, and dNTPs. The prepared paired-end library was created by adapter-ligating the generated double stranded cDNA fragments. For PCR amplification, adaptor-ligated cDNA was utilized. On the Agilent Bioanalyzer 4150 system, library quality was evaluated after PCR products were purified to use the AMPure XP system. Finally, 150 bp paired-end reads were produced using the Illumina Novaseq 6000 (Illumina, San Diego, CA, USA) to sequence the library preparations.

### 2.8. RNA-Seq Data Analysis

On the Illumina platform, RNA-Seq data analysis was carried out. Shanghai Applied Protein Technologies was used for all analyses. These are the main software elements and parameters.

#### 2.8.1. Quality Control

Fastq-format raw data (or raw reads) were initially processed using custom Perl scripts. In this step, the adapter sequence must be removed, followed by the filtering out of low-quality read (low-quality reads are those where the proportion of lines with a string quality value of less than or equal to 25 accounts for more than 60% of the entire reading) and N read (N reads are those where the base information cannot be determined) ratios greater than 5% in order to produce clean reads suitable for further analysis.

#### 2.8.2. Mapping

HISAT2 software (version: 2.1.0, http://daehwankimlab.github.io/hisat2/ format: 29 November 2022.) was then used to align clean reads sequentially to the reference genome in orientation mode to produce mapped reads [23].

#### 2.8.3. Quantification of Gene Expression Level 

The amount of reads that were mapped to each gene was counted using Feature Counts (version: 2.0.0, http://subread.sourceforge.net/ format: 29 November 2022.). The length of each gene and the number of reads mapped to it were used to compute the FPKM of each gene.

#### 2.8.4. Differential Expression Analysis

Differentially expressed genes (DEGs) with |log2FC| > 1 and *p*-value 0.05 were regarded to be significantly different expressed genes. Differential expression analysis was carried out using DESeq2 (version: 1.34.0, format: 29 November 2022.) [24].

#### 2.8.5. Enrichment Analysis

The functional enrichment of differential genes can be explained through the Gene Ontology (GO) and KEGG enrichment analysis of differential genes, which can also shed light on the variations between samples at the level of gene function. For KEGG pathway enrichment analysis and GO function enrichment, we used the cluster Profiler R software package. The GO or KEGG function is deemed considerably enriched when *p* < 0.05 [25,26].

### 2.9. Quantitative Reverse-Transcription PCR(RT-qPCR) Analysis

Using RNA isolater Total RNA Extraction Reagent (Vazyme, Nanjing, China, R401-01) and the FastKing RT kit (with gDNA) (Tiangen, Beijing, China, KR116), spleen tissue from six mice in each group at 100 mg/spleen tissue was extracted from total RNA samples and reverse-transcribed into cDNA. The reverse-transcription reaction conditions were 42 °C for 3 min, 42 °C for 15 s, 95 °C for 30 min, and 0 °C throughout. SuperReal PreMix Plus (SYBR Green) (Tiangen, China, FP205) was used to perform qPCR on the CFX96 System (Bio-Rad, Hercules, CA, USA). The main mixtures were prepared and the cycle conditions were set (predenaturation stage at 95 for 15 min × 1 cycles; PCR reaction stage denaturation at 95 for 15 s; annealing/elongation at 63 for 30 s × 45 cycles; and melting curve stage at 95 °C for 15 s, 60 °C for 60 s, and 95 °C for 15 s × 1 cycles). The sequences of the primers used for the qPCR validation of RNA-Seq Data are listed in Table 1. The 2^−∆∆*CT*^ method was used to compute the relative expressions of genes, and they were then normalized to the Glyceraldehyde-3-phosphate dehydrogenase reference gene (Gapdh).

### 2.10. Statistical Analysis

In this work, all data were collected from at least three different experiments and are expressed as mean ± standard deviation (SD). A two-sample *t*-test was used to statistically analyze differences between the two groups. At a level of *p*-values < 0.05, differences were deemed significant. The experimental data were analyzed and plotted using SPSS 26 and GraphPad 9.0 software.

## 3. Results

### 3.1. Oral Exposure to TDCPP Caused Systematic Response and Induced Splenic Damage

**Figure 1:**
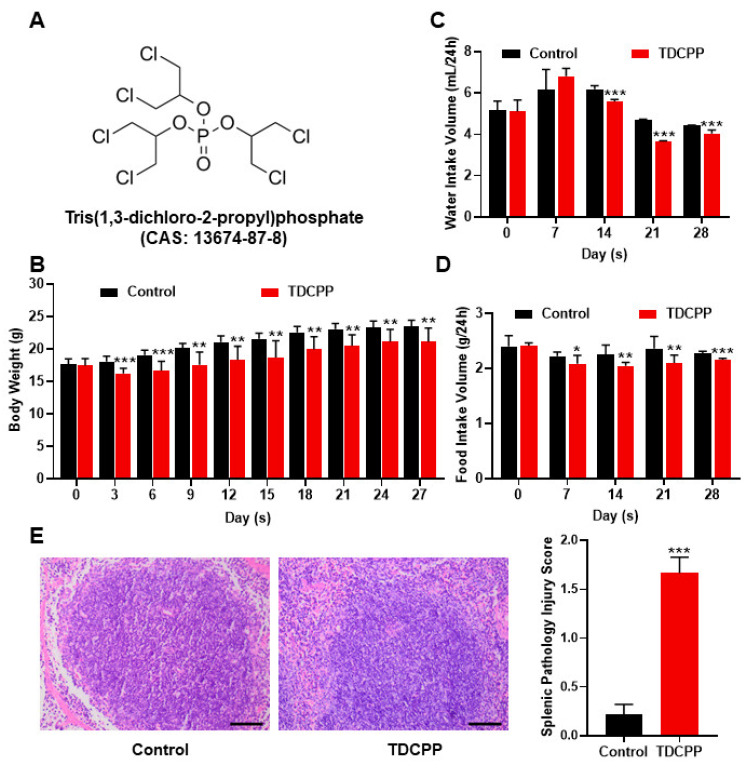
Oral exposure to TDCPP caused systematic response and induced splenic damage. (**A**) Chemical structural formula of TDCPP. (**B**) Body weights of mice in each group were measured on days 0, 3, 6, 9, 12, 15, 18, 21, 24 and 27 under oral administration of 0 or 300 mg/kg TDCPP per day for 28 consecutive days. (**C**) Measurement of 24 h water intake on days 7, 14, 21 and 28 of TDCPP exposure. (**D**) Twenty-four-hour food intake was measured at days 7, 14, 21 and 28 of TDCPP exposure. (**E**) Examination of the effect of TDCPP on spleen histomorphology by fluorescence microscopy of H&E staining of spleen tissue. * *p* < 0.05, ** *p* < 0.01, *** *p* < 0.001 comparing with control group.

In Figure 1A, the chemical formula for TDCPP is displayed. One of the most crucial measures of an animal’s health status is its body weight. Therefore, at the same time of day, we measured the weight of mice in each group on Days 0, 3, 6, 9, 12, 15, 18, 21, 24 and 27 of TDCPP exposure. The body weights of all animals were similar at the beginning before TDCPP exposure. Starting from day 3, compared to the control group, the average body weight of mice in the TDCPP group was significantly lower (Figure 1B). Similarly, animals in the TDCPP group started to have significantly reduced 24 h water and food intake since day 14 and day 7, respectively (Figure 1C,D). In addition, as shown by the H&E staining of spleen tissue (Figure 1E), the white pulp of mice spleen in the TDCPP group was expanded and fused, with a blurry boundary, and the red pulp color was significantly deepened due to vascular expansion. Additionally, there was a significant difference in the splenic pathological damage scores between the two groups of animals, which showed a significantly higher TDCPP than the control group.

### 3.2. Exposure to TDCPP Activated the NF-kB Pathway and Induced Inflammation in the Spleen

**Figure 2:**
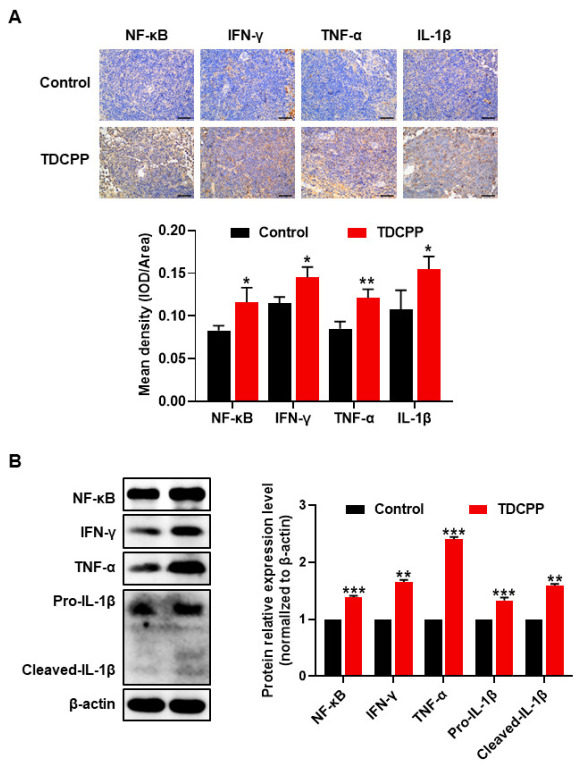
Exposure to TDCPP activated the NF-kB pathway and induced inflammation in the spleen. (**A**) To explore the changes in NF-κB signaling in the inflammatory response to TDCPP-induced splenic tissue injury, NF-κB/IFN-γ/TNF-α/IL-1β expression and tissue localization were detected in mice spleen using IHC. (**B**) The protein expressions of NF-κB/IFN-γ/TNF-α/IL-1β were detected using Western blotting. * *p* < 0.05, ** *p* < 0.01, *** *p* < 0.001, compared with control group.

The NF-κB signaling pathway is involved in physiological and pathological processes, such as infection, immune regulation, inflammatory reaction and tumor formation [27]. Therefore, to investigate the role of potential mechanisms of inflammatory response in splenic tissue injury by TDCPP, we analyzed the protein expression of NF-κB/IFN-γ/TNF-α/IL-1β in mice spleens using IHC and WB, respectively. As indicated by the IHC staining and quantification in Figure 2A, the expressions of NF-κB, IFN-γ, TNF-α and IL-1β were all more highly expressed in the TDCPP group compared to the control group. Similar trends were found by immunoblotting NF-κB, IFN-γ, TNF-α and IL-1β in total protein lysates of spleen tissues from control or TDCPP-treated mice (Figure 2B). IL-1β is a pro-inflammatory cytokine produced mainly by activated monocytes and epithelial cells. Pro-IL-1β is cleaved by caspase-1 into cleaved-IL-1β, which is the mature form of IL-1β and a good indicator of caspase-1 activity. Overall, these results indicate that the subchronicintragastric exposure of TDCPP may trigger an inflammatory response in the spleen through the activation of the NF-κB/IFN-γ/TNF-α/IL-1β pathway.

### 3.3. TDCPP-Induced Apoptotic Cell Death in the Spleen

**Figure 3:**
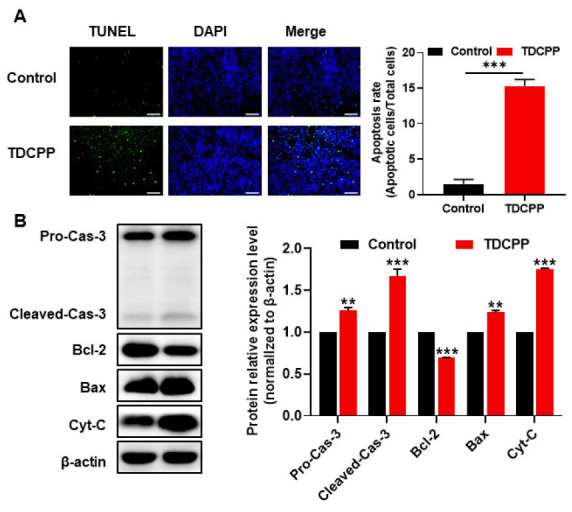
TDCPP-induced apoptotic cell death in the spleen. (**A**) The spleens of both groups of mice were examined by TUNEL assay, and the apoptotic rates of TUNEL-positive cells were calculated. (**B**) Detection of caspase-3/Bcl-2/Bax/Cyt-C protein expression levels using Western blotting to determine that TDCPP exposure leads to apoptosis in mice spleen tissue via the mitochondrial apoptosis pathway. ** *p* < 0.01, *** *p* < 0.001, compared with control group.

TDCPP was shown to increase the activities of caspase-3 and caspase-9, promote the expression of Bax, inhibit the expression of Bcl-2, and finally lead to apoptosis through the mitochondrial apoptosis pathway [1]. Apoptosis can also be induced through the activation of death receptors, such as the interaction of TNF-α with TNF-αR [28]. As shown in Figure 3A, the proportion of TUNEL-positive cells in the spleen tissues from the TDCPP group was significantly higher than that of the control group. Consistent with the TUNEL staining, pro-caspase 3 and cleaved-caspase 3 expressions were increased in the TDCPP group in comparison to the control group. The expression of BAX and cytochrome C was also increased in TDCPP group. Further, there was a decrease in Bcl-2 expression in the TDCPP group. The above results show that TDCPP exposure leads to the apoptosis of mice spleen tissue cells via the mitochondrial apoptotic pathway.

### 3.4. RNA-Seq Analysis of Mice Spleen Tissue

**Figure 4:**
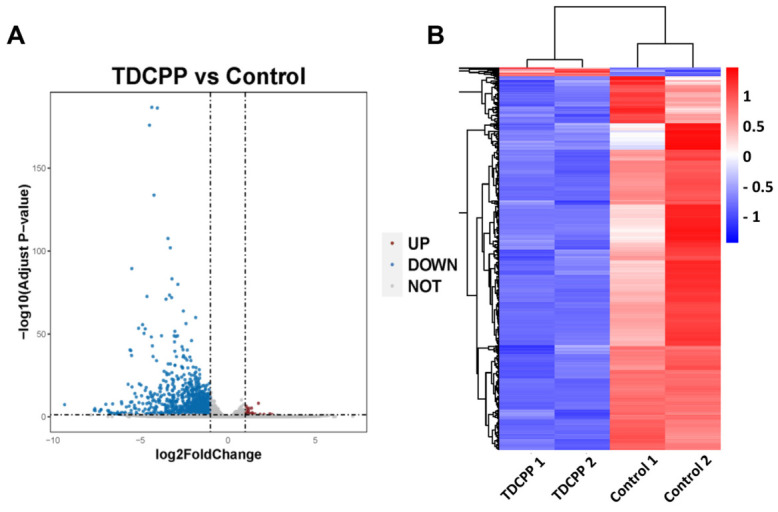
RNA-Seq analysis of mice spleen tissue. (**A**) DEGs were analyzed and volcano plots were drawn for mice treated with different doses of TDCPP. (**B**) Hierarchical clustering heat maps were plotted for DEGs between the control and TDCPP groups, and increases in expression are indicated in red, while decreases are indicated in blue.

To get a broader picture of the changes post TDCPP exposure in the spleen at the transcriptome level, we performed RNA-seq analysis on RNAs isolated from the spleens. A total of 47,708,220 genes were identified, 97.29% of the sequence bases could be aligned to the genome, and the junction reads added up to more than 27.71% (Table S1). Gene expression levels were calculated for each sample in both groups, and the overall distribution of sample gene expression and inter-sample correlation were analyzed. As a result, 1025 DEGs in mice spleen were identified after TDCPP stimulation (Table S1). Overall, 23 upregulated and 1002 downregulated DEGs were identified in the TDCPP vs. control groups, as is shown in the DEG volcano plot (Figure 4A), statistical table of differential gene expression (Table S2), and DEG cluster heatmap (Figure 4B). 

### 3.5. GO Analysis

**Figure 5:**
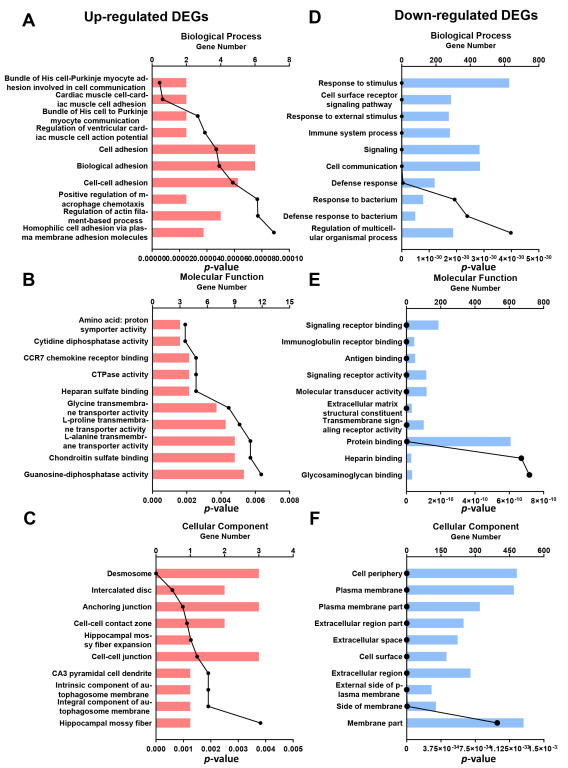
GO analysis of DEGs in TDCPP vs. control. The top 10 most significant *p*-values for the GO terms were upregulated by TDCPP compared to controls in terms of biological processes (BP) (**A**), molecular functions (MF) (**B**), and cellular components (CC) (**C**). The top 10 most significant *p*-values for the GO terms were downregulated by TDCPP compared to controls in terms of biological processes (BP) (**D**), molecular functions (MF) (**E**) and cellular components (CC) (**F**).

To identify the function of DEGs in the spleen of mice after TDCPP exposure, a GO enrichment analysis of DEGs was carried out based on the GO database. The results show the top 10 GO terms according to secondary classification terms, biological process (BP), molecular function (MF) and cellular component (CC). In the BP annotation category, the upregulated genes were mainly associated with cell adhesion, while the downregulated genes were enriched for the immunologic process in the TDCPP vs. control groups (Figure 5A–C). In the MF and CC categories, the upregulated genes were in the intracellular junctional components, while the downregulated genes were enriched for cell membrane components in the TDCPP vs. control groups (Figure 5D–F). 

### 3.6. KEGG Enrichment Analysis

**Figure 6:**
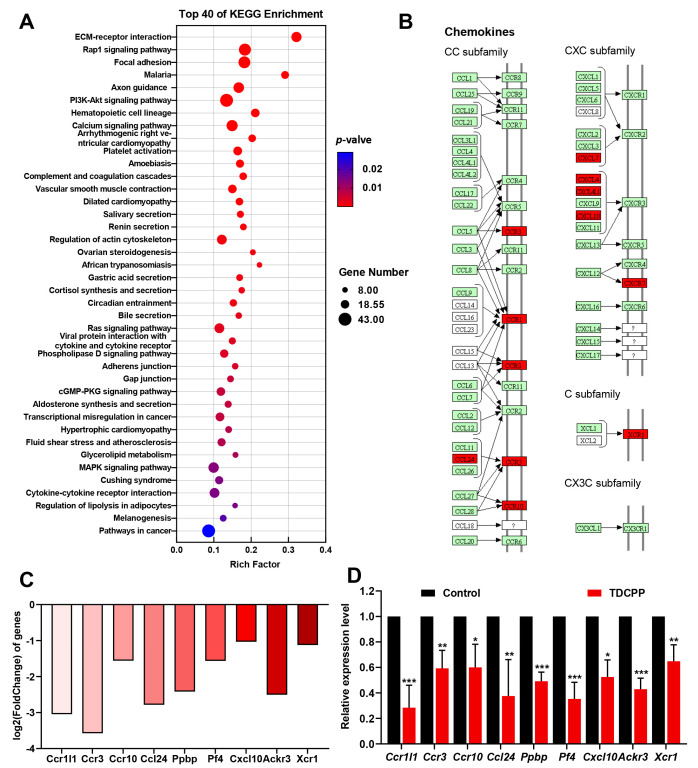
KEGG enrichment analysis and validation of gene expression of TDCPP vs. control DEGs. (**A**) The most significant *p*-values for the KEGG enrichment pathways were up- and downregulated by TDCPP vs. control. (**B**) Gene expression of chemokines in the cytokine–cytokine receptor interaction pathway. Red markers indicate downregulated genes. (**C**) The RNA sequencing results showed that the DEGs of chemokines in the cytokine–cytokine receptor interaction pathway in spleen tissue of TDCPP vs. control groups. (**D**) Validation of DEGs of chemokines in the cytokine–cytokine receptor interaction pathway in splenic tissues of TDCPP vs. control groups through qRT-PCR. * *p* < 0.05, ** *p* < 0.01, *** *p* < 0.001, compared with control group.

We found 264 markedly impacted metabolic and signaling pathways using the KEGG pathway as a unit and the reference genome as the background. The KEGG enrichment pathway includes the top 40 up- and downregulated genes, as shown in Figure 6A, including: environmental information processing (signal transduction, signaling molecules and interaction), cellular processes (cellular community–eukaryotes, transport and catabolism, cellular community–eukaryotes), human diseases (infectious disease: parasitic, cardiovascular disease, environmental adaptation, cancer: overview, immune disease, infectious disease: bacterial), organismal systems (development and regeneration, immune system, digestive system, circulatory system, endocrine system), and metabolism (lipid metabolism, xenobiotics biodegradation and metabolism).

To further explore the effects of TDCPP on splenic immune and inflammatory responses, we noted the following ten KEGG pathways: complement and coagulation cascades, hematopoietic cell lineage, platelet activation, chemokine signaling pathway, neutrophil extracellular trap formation, ECM-receptor interaction, viral protein interaction with cytokine and cytokine receptor, cytokine-cytokine receptor interaction. Chemokines play a key role in inflammation and immunity by interacting with receptors to regulate the targeted migration of immune cells, remove sources of infection, and promote wound healing, among other functions [29]. Therefore, to investigate the effect of TDCPP on the spleen, we chose the cytokine–cytokine receptor interaction pathway for validation (Figure 6B,C). We validated nine candidate genes for the cytokine–cytokine receptor interaction pathway, including: Ccr1l1, Ccr3, Ccr10, Ccl24, Ppbp, Pf4, Cxcl10, Ackr3, and Xcr1.

### 3.7. Validation of Gene Expression

As shown in Figure 6D, TDCPP significantly inhibited the expressions of Ccr1l1, Ccr3, Ccr10, Ccl24, Ppbp, Pf4, Cxcl10, Ackr3, and Xcr1 mRNAs in splenic tissue transcriptome sequencing, demonstrating the ability of TDCPP to inhibit the cytokine–cytokine receptor interaction pathway. As noted, we validated nine candidate genes. Consistent with RNA-Seq data, according to qRT-PCR analysis, compared to the control group, the expressions of chemokine-related genes, Ccr1l1, Ccr3, Ccr10, Ccl24, Ppbp, Pf4, Cxcl10, Ackr3, and Xcr1 in spleen tissue were significantly reduced (*p* < 0.05) (Figure 6D). 

## 4. Discussion

TDCPP has been widely used in fire-susceptible items as one of the main PBDE alternatives over the past 20 years [1]. TDCPP, however, could leak out of items into different environmental media over the course of their lifetimes, in the same way that PBDEs do. For instance, through the industrial processing of food products, TDCPP entered food and was eaten by humans as a result [30]. Over the past few decades, studies on the toxicological effects of this product have mainly focused on reproduction [31], development [32], nerve toxicity [33], and endocrine disruption [34]. Research on immune system damage is still unclear, especially regarding the mechanisms of injury. In the present study, we employed environmental toxicology and transcriptome analysis techniques to determine the immunotoxicity of TDCPP exposure to mice spleen. As the largest secondary immune organ in our body, the spleen is an important line of defense for organisms against disease and invasion by harmful foreign bacteria and viruses, and it is susceptible to environmental perturbations. The spleen histomorphology of TDCPP-treated mice showed pathological changes. These pathomorphological alterations in the spleen are similar to previous findings, which showed that BDE-209 suppresses splenic cell immune and physiological functions by inducing inflammation and apoptosis, ultimately leading to splenic atrophy [35] and cadmium-induced spleen toxicity in mice [36], suggesting that these environmental pollutants cause splenic toxicity in a similar way. Body weight is one of the important indicators of the health of organisms and is influenced by several factors, such as excessive or insufficient energy intake and abnormal energy consumption, such as excessive exercise and disease. Therefore, when monitoring the weight of each group of mice, we also monitored their 24 h water intake and food intake every 7 days, respectively. After excluding the effects of drinking and feeding from the i.g operation, we found that TDCPP treatment might inhibit drinking and feeding in mice. This might be one of the main reasons for the weight change of TDCPP mice in a short time after treatment. Similarly, Sun et al. also noted in their study that rapid weight loss is primarily caused by a decrease in energy intake and not by an increase in energy expenditure or cachexia [37].

The inflammatory cytokine TNF-α can promote the inflammatory response by activating the NF-κB signaling pathway and further regulating the expression of downstream interleukin-related genes, adhesion factor-related genes, and other genes [38,39,40]. Our results are supported by previous reports of increased expression of NF-κB, TNF-α, and IL-1β in splenic tissue after TDCPP treatment, which further leads to splenic inflammation [39]. Moreover, some studies have also carried out in vivo and ex vivo tests that showed that TDCPP can increase the expressions of the pro-inflammatory factors Il-1beta and Tnf-mRNA in hippocampus neuronal cells, resulting in the induction of neurological inflammation [13]. IFN-γ is a cytokine with immune-regulatory, antivirus, antitumor, and antiparasitic effects, and it plays an essential role in maintaining the cellular immunity of the body, secreted by activated NK cells and T cells [41]. In a mouse model of aplastic anemia induced by irradiation and spleen–thymus lymphocyte infusion, the levels of the cytokines IL-6, IL-8, IL-17, TNF-α, and IFN-γ were increased in peripheral blood and bone marrow, and an immunoinflammatory response occurred [42]. Interestingly, this is consistent with our finding of increased IFN-γ and TNF-α expression in the spleen when the spleen was subjected to TDCPP. The above evidence suggests that TDCPP-induced inflammatory responses in the spleen are accompanied by the activation of the NF-κB signaling pathway. 

In addition, TDCPP caused apoptosis by altering the transcriptional levels of bcl-2, bax, and caspase 3 genes, which ultimately led to apoptosis in SH-SY5Y cells [13]. In normal human-skin keratinocytes, TDCPP caused the protein expression of D1, CDK2, CDK6, and Bcl-2, in addition to boosting Bax and Caspase-3 expression to cause apoptosis and cell cycle arrest [43]. Corresponding to this, in our study, we discovered that in the spleen tissues of mice given TDCPP, the relative expressions of caspase 3, BAX, and cytochrome C proteins were elevated, whereas the relative expressions of Bcl-2 proteins were lowered. Thus, the activation of mitochondrial apoptosis pathways is important for TDCPP-induced cytotoxicity. It is worth noting that some studies suggest that the impairment of splenic immune function may be via immunosuppression and the activation of the NF-B signaling pathway [44,45].

According to the GO analysis, the DEGs were mainly related to the immunologic process, metabolic process, and cellular components. Therefore, these results are almost consistent with our conjecture that TDCCP induces immunoinflammatory toxicity in mouse spleen. It also provides a foundation for the selection of candidate genes to further study the molecular mechanism of immunoinflammatory-related toxicity of TDCPP in the spleen. In addition, the KEGG pathway analysis identified eight pathways related to immunoinflammatory responses. Considering the purpose of this study and the number of differentially enriched genes, we selected the cytokine–cytokine receptor interaction pathway as the subsequent point of interest. We only focused on nine candidate genes for the cytokine–cytokine receptor interaction pathway, including four genes of the CC subfamily, four genes of the CXC subfamily, and one gene of the C subfamily. The development and homeostasis of the immune system depend on chemokines, which are involved in all immunological and inflammatory responses, whether they are protective or damaging [46]. CD4+ and CD8+ lymphocytes, dendritic cells, eosinophils, macrophages, monocytes, and NK cells are immune system cells that depend on CC family chemokines for survival [47], as does tumor development. In individuals with immunological thrombocytopenia, the expression of the Th1- and Th2-associated chemokine CCR3 gene was reduced [48], and animals lacking the chemokine receptor CCR1 had elevated Th1 responses and glomerular damage in nephritis [49]. In addition, CCL24 can combine with CCR3 to recruit eosinophils and tumor-associated macrophages, and immune function is limited when CCR3/CCL24 is suppressed [50]. Moreover, research has demonstrated that CXC chemokines either promote or inhibit immunity, which in turn affects the development of cancer [51]. The XCL1-XCR1 axis plays an important role in ensuring effective CD8 T cell-mediated cytotoxic immune responses, such as the ability of XCR1 to promote the CD8 DC activation of early CD8 T cell-mediated defense against intracellular pathogenic bacteria [52]. In this study, TDCPP significantly inhibited Ccr1l1, Ccr3, Ccr10, Ccl24, Ppbp, Pf4, Cxcl10, Ackr3, and Xcr1 genes expression in spleen tissue homogenates according to RNA-Seq and qRT-PCR, which indicates that the cytokine–cytokine receptor interaction pathway is a critical toxic target of TDCPP.

## 5. Conclusions

In summary, by combining cell biological assessment and transcriptome analysis techniques, we revealed the gene expression changes that were affected by TDCPP. First, mice treated with TDCPP i.g for 28 consecutive days were found to have altered general conditions and induced pathological changes in the spleen. Second, analysis of relevant inflammatory factors in splenic tissues showed that TDCPP can activate mitochondrial apoptotic pathways and alter apoptosis-related proteins. Third, RNA-seq analysis showed that TDCPP induced changes in eight KEGG pathways associated with immune and inflammatory responses in the spleen. Subsequently, qRT-PCR was used to validate 17 genes of the cytokine–cytokine receptor interaction pathway and revealed that TDCPP caused immunosuppression and induced an inflammatory response to toxins in the spleen. Accordingly, our study confirmed the immunotoxicity of TDCPP exposure to mice characterized by an inflammatory reaction, the activation of mitochondrial apoptosis pathways, and the inhibition of the expression of chemokines and their related receptors, causing an immunosuppressive effect.

## Figures and Tables

**Table 1:** Primer sequences for the qPCR validation of RNA-Seq data.

Gene Name	Gene ID	Primer Sequence (5′–3′)	Tm (°C)	Length (bp)
Chemokine				
CC subfamily				
Ccr1l1	12770	GGCATCATCAGTAGCATCA	55	303
		GAGGAAGAAGAGAAGCGTAA		
Ccr3	12771	GCTTTGAGACCACACCCTATG	58	136
		ACCATTATGTTGCCCAGGAG		
Ccr10	12777	ATGCTTCACTCGGTCTCT	55	127
		CACTACTGGATAGCGATAGG		
Ccl24	56221	GGTTCAGAGGCACATACAA	55	492
		AGAGATGGACAGACAGACA		
CXC subfamily				
Ppbp	57349	TGTGCTGATGTGGAAGTGATAG	58	150
		CTGAGCAGGAAAATGGTTTGG		
Pf4	56744	GAGGTGATCAAGGCAGGAC	58	141
		AGCTGATACCTAACTCTCCAGG		
Cxcl10	15945	GATGGATGGACAGCAGAG	54	417
		GGAAGATGGTGGTTAAGTTC		
Ackr3	12778	TTCATCAACCGCAACTACA	55	260
		TCTCCTCTTCATACCACTCA		
C subfamily				
Xcr1	23832	ATCTTCTTCCTCCTCTCCAT	55	473
		ATCCACTTCTCCTTGTCTTC		
**Glyceraldehyde-3-phosphate dehydrogenase protein family**				
Gapdh	14433	TCTCCTGCGACTTCAACA	56	117
		TGTAGCCGTATTCATTGTCA		

## Data Availability

The data provided in this study are available upon request from the corresponding authors. Due to privacy issues, these data cannot be made public.

## Supplementary Materials

The following supporting information can be downloaded at: https://www.mdpi.com/article/10.3390/toxics11030231/s1, Table S1: Statistics of Reads and Reference Genome Comparison; Table S2: Statistical Table of Differential Gene Expression.
